# Malignant lung PEComa (clear cell tumor): rare case report and literature review

**DOI:** 10.3389/fonc.2023.1260844

**Published:** 2023-09-20

**Authors:** Marcos Adriano Garcia Campos, Lucas Fernandes Vasques, Rafael Goulart de Medeiros, Érico Murilo Monteiro Cutrim, Ana Júlia Favarin, Sarah Rebecca Machado Silva, Gyl Eanes Barros Silva, Marcelo Padovani de Toledo Moraes, Mariana Lopes Zanatta, Diego Aparecido Rios Queiróz

**Affiliations:** ^1^ Department of Internal Medicine, Hospital of Medical School of São Paulo State University, Botucatu, Brazil; ^2^ Department of Medicine, University of Taubate Faculty of Medicine, Taubaté, Brazil; ^3^ Laboratory of Immunofluorescence and Electron Microscopy, University Hospital of Federal University of Maranhão, São Luís, Brazil

**Keywords:** PEComa, sugar tumor, pulmonary cancer, cathepsin K, HMB-45

## Abstract

Clear cell tumors of the lung (CCTL), or “sugar tumors” of lung, are very uncommon lesions and are mostly benign perivascular epithelioid cell (PEC) tumors with no specific morphologic features. Fewer than 100 cases have been reported; the aggressive nature demonstrated in sporadic reports has rarely been described in the literature. Although the course is generally described as benign, eight reported cases showed malignant behavior. We report a case of a PEC with a malignant presentation in a young man, correlating the main characteristics of the tumor with other cases reported in the literature to better elucidate this rare presentation. We also performed a literature review of reports on benign and malignant CCTL cases, with a focus on clinical, imaging, and immunohistochemical differentiation. CCTLs are rare tumors that require histopathological and immunohistochemical confirmation; to date, criteria that can predict malignant evolution are lacking.

## Introduction

Clear cell tumors, or “sugar tumors”, are very uncommon lesions (1) and are mostly benign perivascular epithelioid cell (PEC) tumors (2, 3). PEC tumors are a rare group of mesenchymal neoplasms histologically and immunohistochemically distinct from other neoplasms, with an abundance of periodic acid-Schiff (PAS)-positive glycogen (3). They are expressed in the bladder (4), uterus (5), breast (6), ovary (7), vagina (8), kidney (9), prostate (10), pancreas (11), jejunum (12), soft tissues (13) and lung (14). Due to this wide diversity, these tumors lack specific morphologic features (2).

The lungs are an uncommon site for PEC tumors, known as clear cell tumors of lung (CCTL) (15), and such PEC tumors in the lung are usually benign, with malignancy exceedingly rare (9). Although fewer than 100 cases have been reported, sporadic reports have described CCTLs with very rare aggressive nature (3). A silent, indolent, and asymptomatic presentation may pose a diagnostic challenge for pathologists and oncologists (16).

CCTLs exhibit nested histological features similar to other PEC tumors. They consist of uniform round to polygonal epithelioid cells with clear or eosinophilic cytoplasm and well-defined borders. CCTLs are surrounded by prominent thin-walled vascular channels. Although the course of CCTL is typically described as benign, eight cases have shown malignant behavior (17). We present a case of CCTL with a malignant presentation, correlating the main characteristics of the tumor with other cases reported in the literature to better elucidate this rare presentation.

## Case description

A 49-year-old man, hypertensive and a smoker, presented to the primary healthcare department with complaints of dyspnea on exertion and daily chest pain for the past year, with gradual worsening in the last three months, in addition to a weight loss of 10 kg. The patient denied coughing, hemoptysis, fever, or a family history of cancer.

**Figure 1:**
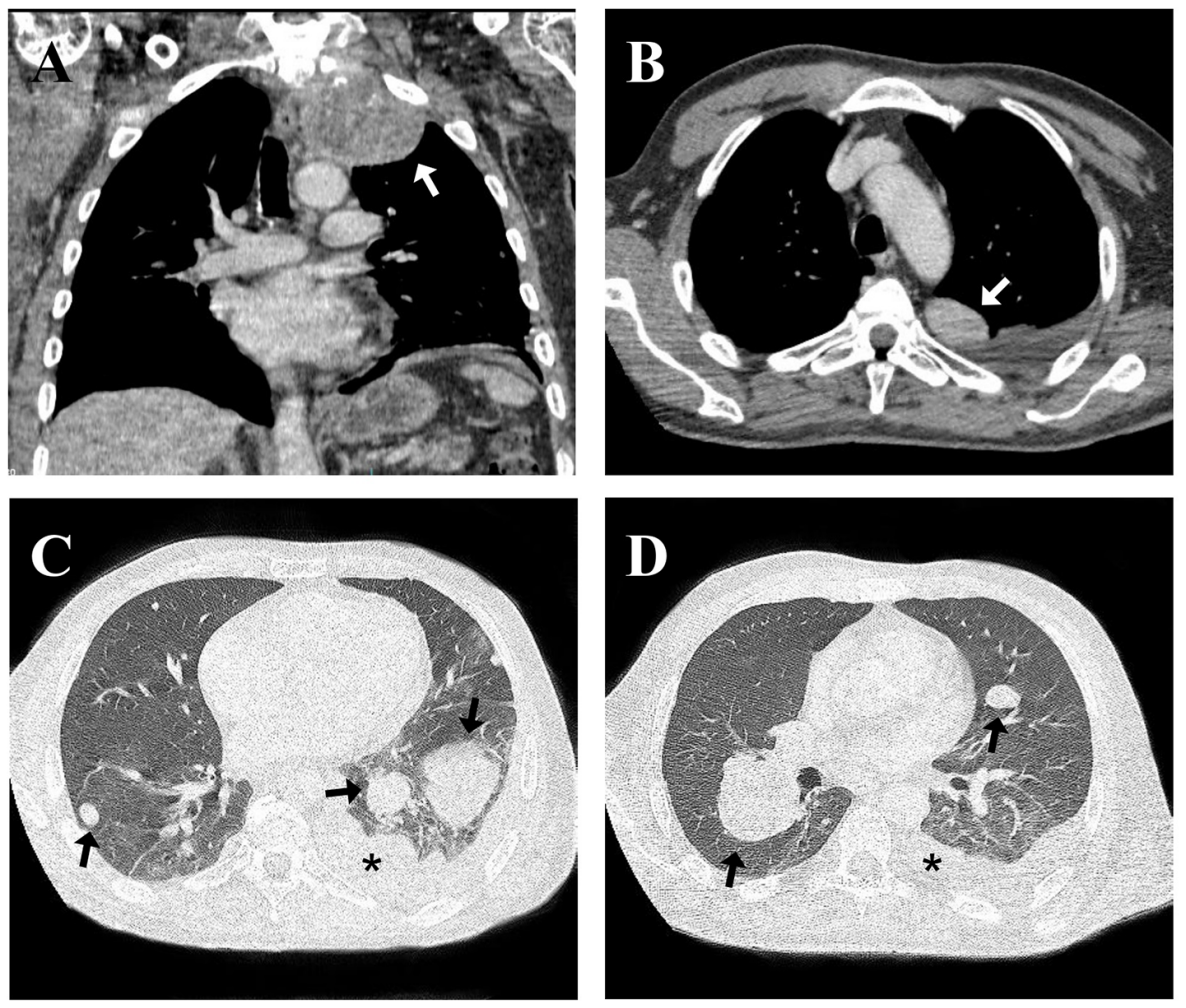
Initial coronal **(A)** and axial **(B)** computed tomography pulmonary angiogram reconstruction of the chest performed showing a mass in upper left paratracheal side (white arrow in **A, B**) associated with multiple randomly distributed nodules in the lung parenchyma (black arrow in **C, D**), the largest of them in the middle third of the lung right, enlarged lymph nodes in the para-aortic chain and thin left pleural effusion (black asterisk in **C, D**).

Computed tomography (CT) of the chest showed a solid, calcified, upper left paratracheal lesion (191 cm^3^) with central necrosis in close contact with the ipsilateral subclavian artery without a cleavage plane, in addition to multiple randomly distributed nodules in the lung parenchyma and enlarged lymph nodes in the para-aortic chain and thin left pleural effusion (**Figure 1**). A lithic image of the T1 vertebral body indicated possible secondary neoplastic involvement; however, bone scintigraphy and positron emission tomography-CT (PET-CT) revealed a low probability of bone metastasis. No secondary lesions were observed in any of the abdominal organs.

**Figure 2:**
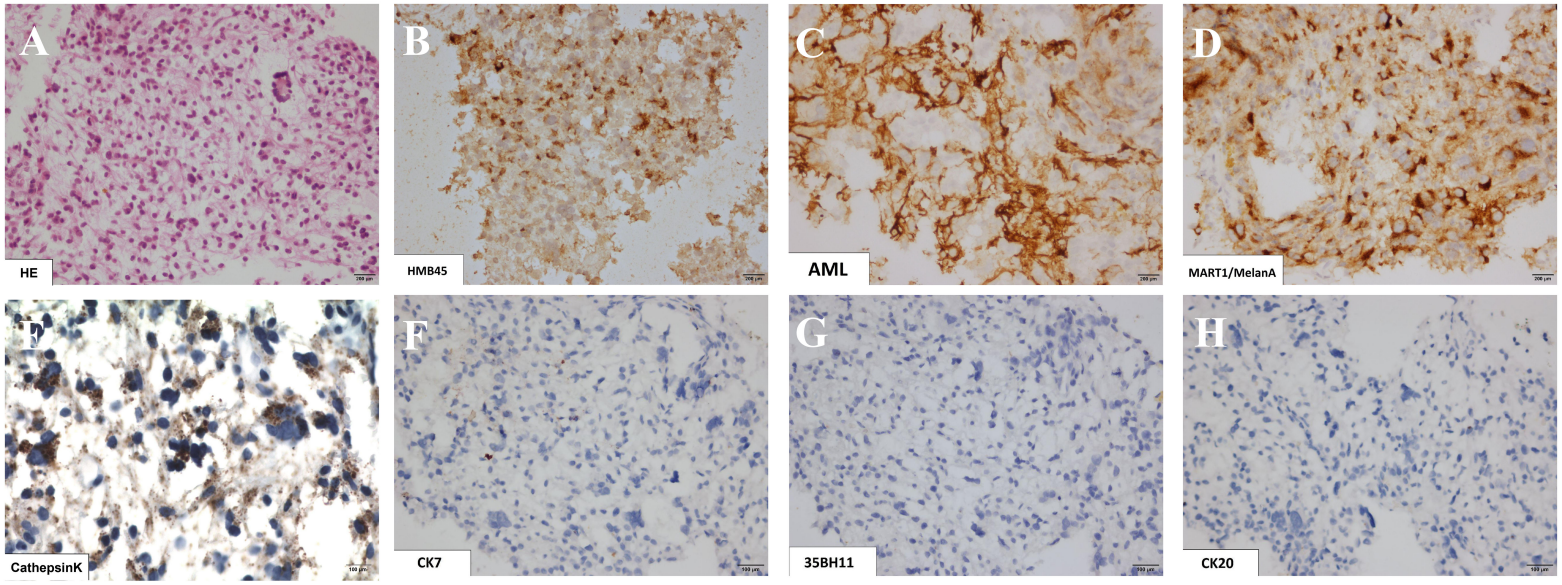
Histological sections of lung biopsy showing in hematoxylin-eosin staining the presence of clear cell tumors represented by epithelioid cells with abundant clear and granular cytoplasm, with well-delimited cell borders and round and hyperchromatic nuclei, discreet cell atypia and absence of significant mitotic activity **(A)**. The immunohistochemical study revealed co-expression of markers: HMB45 **(B)**, smooth muscle actin **(C)**, Melan-A **(D)** and Cathepsin K **(E)**, and negativity for cytokeratins CK7, CK8 and CK20 (**F**, **G**, **H**, respectively).

The anatomopathological examination of the transbronchial biopsy showed the presence of neoplasia caused by epithelioid cells with abundant clear and granular cytoplasm, well-defined cell borders, and round and hyperchromatic nuclei, without the presence of prominent nucleoli (**Figure 2**). There were mild cellular atypia and foci of hemorrhage, necrosis, and absence of significant mitotic activity. Immunohistochemical (IHC) analysis revealed negativity for pan-cytokeratin (AE1, AE3, TTF1, RCC, MiTF, S-100, CK7, CK8, CK20, and desmin) and positivity for muscle markers and melanocyte antibodies (HMB-45, smooth actin muscle, CD10, and Melan-A) and Cathepsin K. These findings were indicative of a CCTL.

The challenges of verifying diagnosis and scheduling medical appointments, combined with suboptimal patient adherence, led to delayed treatment initiation. Only, five months after the biopsy, the patient underwent 2000 cGy doses of radiotherapy targeting the left lung in five fractions over one week and three cycles of doxorubicin 75 mg/m^2^ within three months. However, owing to his compromised performance status and the presence of multiple nodules, surgical resection could not be performed. He presented with deterioration of the clinical picture and progression of pulmonary nodules in number and size two months after starting treatment, in addition to an increase in mediastinal lymph nodes and bilateral pleural effusion.

**Figure 3:**
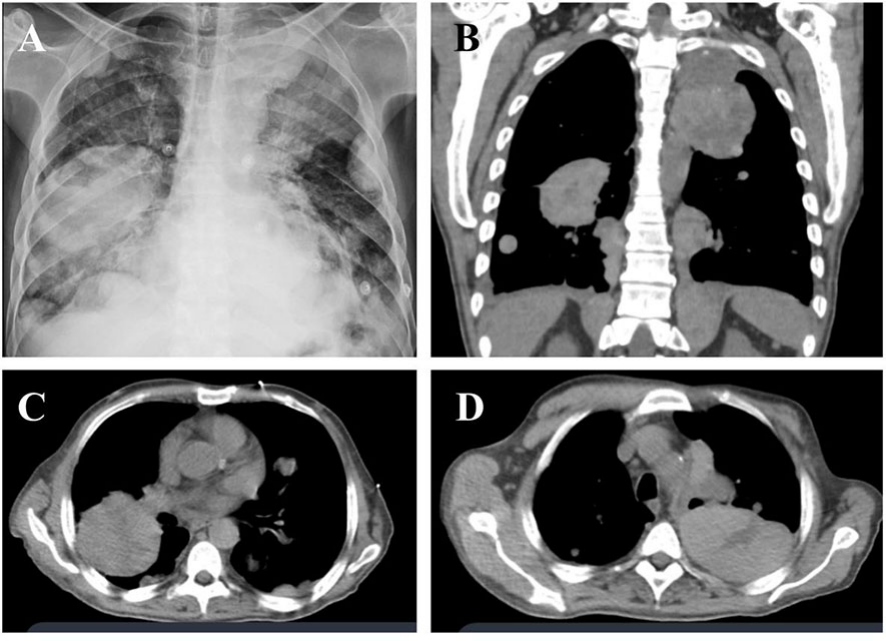
Radiographic images showing extensive pulmonary masses on chest X-ray **(A)** and computed tomography of the chest in coronal **(B)** and axial planes **(C, D)**, suggesting disease progression. A solid tumor, calcified upper left parasternal lesion with central necrosis, close contact with ipsilateral subclavian artery, without a cleavage plane and a tumor with similar characteristics on the lower right.

**Figure 4:**
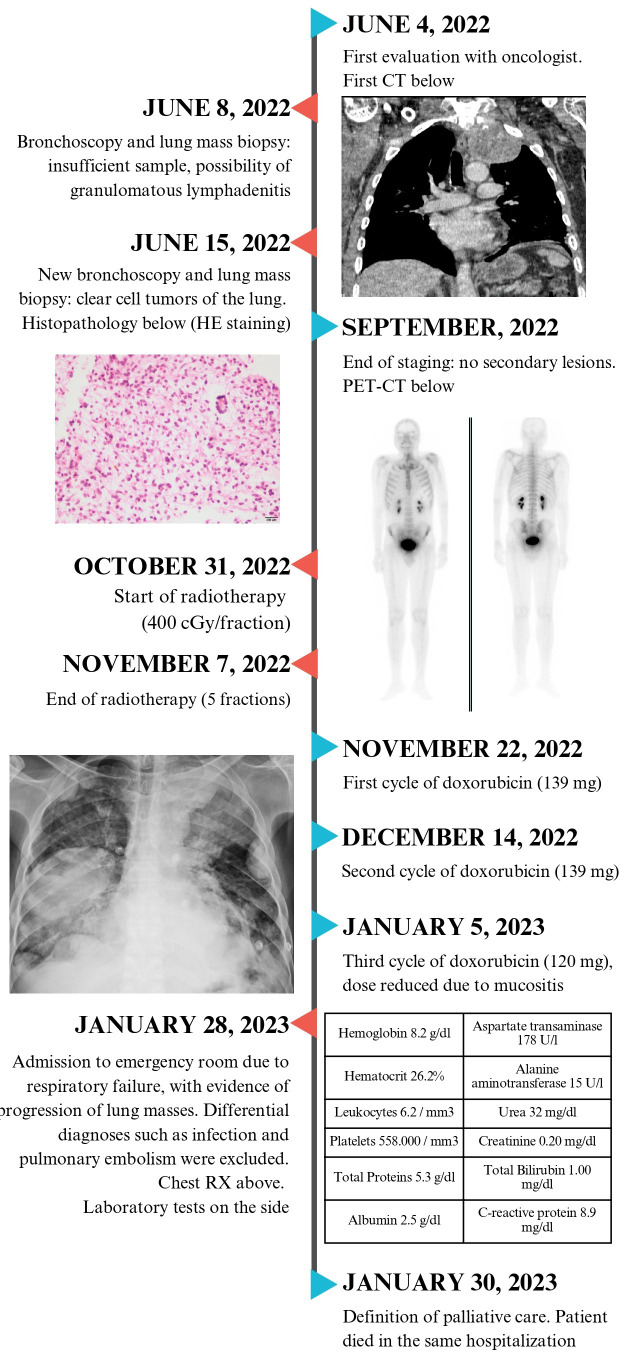
Timeline from the initial clinical presentation and tests performed for the diagnosis of clear cell tumors of the lung. The time of chemotherapy, radiotherapy and hospitalization until the outcome of the case are also specified.

One month later, the patient was admitted to the emergency room with dyspnea, desaturation, and severe chest pain. Additionally, he had mild respiratory alkalosis associated with microcytic and hypochromic anemia without leukocytosis or normal platelet levels. He had normal electrolyte and renal function, increase in transaminase levels by about three times the upper normal value, and slight hypoalbuminemia, without changes in bilirubin or coagulogram. C-reactive protein was 8.9 mg/dl (reference value, < 1 mg/dl), with no reports of infectious symptoms. Serological tests for hepatitis, HIV and syphilis were negative. Chest radiography and CT revealed a significant progression of the lung masses (**Figure 3**). There was no evidence of other complications such as massive pleural effusion, pulmonary embolism, or pneumothorax, which would justify respiratory worsening in addition to the progression of tumor masses. Owing to the rapid progression of his condition, the patient underwent palliative sedation and died approximately 48 h after admission. A timeline specifying the entire clinical evolution of the patient is shown in **Figure 4**.

## Discussion

Herein, we present a rare case of malignant CCTL. It is an uncommon and usually benign neoplasm composed of uniform round to polygonal epithelioid cells with clear or eosinophilic cytoplasm, well-defined borders, and prominent thin-walled vascular channels (1), as observed in our case. Throughout the clinical and imaging evaluation, the malignant presentation of the patient was clear.

CCTL has no established epidemiology, clinical characteristics, diagnoses, or treatments. It is commonly confused with lung carcinoma or metastases from clear-cell carcinoma of the kidney (18). Patients are usually > 50 years old, but cases from 9 to 75 years of age have been reported (15, 19), with a similar prevalence between the sexes (20).

Among the benign manifestations of CCTL, an asymptomatic condition with an accidental diagnosis is common. However, as the size of this neoplasm can vary from several millimeters to approximately 16 cm (15), symptoms such as chest pain, cough, shortness of breath, hemoptysis, or fever may be observed in larger masses, especially if the size exceeds 2.2 cm (21–24).

We performed a literature review of the cases of benign and malignant CCTL (**Table 1**). Cases of CCTL with malignant behavior during the course of the disease and its outcomes have been reported sporadically. Certain characteristics, such as diameter greater than 2.5-3.0 cm, presence of symptoms, metastases, extensive necrosis, and abundant mitoses, are associated with a malignant presentation (24). Of the 76 benign occurrences found, necrosis was present in only 6.5% of the cases and mitotic activity was present in 4.0%. The largest mean mass diameter was 2.8 cm. In malignant cases, necrosis and mitotic activity were significantly more frequent, occurring in 50% and 12.5% respectively, and the mean diameter of the mass was 6.7 cm, which is 2.3 times greater than that of benign masses.

**Table 1:** Comparison of features between benign and malignant cases of perivascular epithelioid cells (PEC) tumors.

Characteristic	Benign (81)^a^ n (%)	Malignant (8)^b^ n (%)
Sex		
Male	45 (55.5)	3 (37.5)
Female	36 (44.4)	5 (62.2)
**Range age**	49 (10 – 75) years	60 (49 – 75) years
Symptoms		
Not reported	31 (38.2)	2 (25.0)
Cough	7 (8.6)	3 (37.5)
Chest pain	13 (16.0)	2 (25.0)
Dyspnoea	8 (9.8)	0 (0.0)
Absente	26 (32.0)	0 (0.0)
Pulmonary tumor site		
Lower right	16 (19.7)	2 (25.0)
Lower left	22 (27.1)	4 (50.0)
Upper left	14 (17.2)	0 (0.0)
Superior right	13 (16.0)	1 (12.5)
Middle right	6 (7.4)	1 (12.5)
Middle left	4 (4.9)	0 (0.0)
**Range of the largest diameter of the lesion**	2.78 (0.4 - 12) cm	6.7 (3 - 16.2) cm
Concurrent nodules		
Single node	75 (92.5)	5 (62.2)
Two nodules	2 (2.4)	1 (12.5)
More than two nodules	4 (4.9)	2 (25.0)
Borders		
Regular	65 (80.2)	5 (62.2)
Irregular	4 (4.9)	3 (37.5)
Comorbidities		
Not reported	9 (11.1)	3 (37.5)
COPD	4 (4.9)	0 (0.0)
SAH	8 (9.8)	0 (0.0)
Dyslipidemia	3 (3.7)	0 (0.0)
Essential Thrombocythemia	2 (2.4)	0 (0.0)
Absente	46 (56.7)	5 (62.2)
Smoking		
Not reported	33 (40.7)	2 (25.0)
Present	19 (23.4)	0 (0.0)
Absent	29 (35.8)	6 (75.0)
Biomarkers		
HMB45	48 (59.2)	5 (62.2)
Vimentin	21 (25.9)	4 (50.0)
S-100	22 (27.1)	3 (37.5)
Melan-A	15 (18.5)	3 (37.5)
CD34	11 (13.5)	1 (12.5)
NSE	11 (13.5)	0 (0.0)
SMA	4 (4.9)	1 (12.5)
CD56	2 (2.4)	1 (12.5)
Necrosis		
Not reported	25 (30.8)	1 (12.5)
Present	5 (6.1)	4 (50.0)
Absent	51 (62.9)	3 (37.5)
Mitotic activity		
Not reported	16 (19.7)	1 (12.5)
Present	3 (3.7)	1 (12.5)
Absent	62 (76.5)	6 (75.0)
Vascularization		
Not reported	18 (22.3)	–
Present	55 (67.9)	6 (75.0)
Absent	8 (9.8)	2 (25.0)

COPD, chronic obstructive pulmonary disease; SAH, systemic arterial hypertension.

a References (1, 3, 18, 19, 21, 24–78).

b References (14, 17, 23, 36, 74–77).

The site that was most affected by the primary nodule was the lower lobe of the lung. In this case, an extensive lesion was observed in the left upper lobe, characterized by necrosis and abundant vascularization, in addition to other scattered nodules in the parenchyma.

CCTL has already been associated with other diseases, such as tuberous sclerosis complex (79), Birt-Hogg-Dube Syndrome, and essential thrombocythemia (26, 27). However, in the malignant cases, it was not possible to determine the presence of risk factors strongly related to CCTL (**Table 2**).

**Table 2:** Malignant cases of perivascular epithelioid cells (PEC) tumors reported in literature.

Reference	Sex/Age	Diagnostic exam	Treatment	Survival
Gaffey M.J. 1990 (36)	Male/66	unreported imaging exam Excisional biopsy	Surgical resection	Metastatic lesions remaining after 13 years. Death after 4 years of recurrence
Jeremy R.P. 2006 (77)	Female/53	CT Excisional biopsy Immunohistochemistry	Surgical resection Resection of brain metastases	Not reported
Ting Ye 2010 (76)	Female/50	CT Excisional biopsy Immunohistochemistry	Surgical resection	Not reported
Benedict Yan 2011 (23)	Female/75	CT Excisional biopsy Immunohistochemistry	Surgical resection	No signs of recurrence after 10 years. Death without clear cause
Hyun-ju Lim 2013 (14)	Male/60	PET/CT Excisional biopsy Immunohistochemistry	Surgical resection	Metastatic lesions remaining after 1 year
Yinghua Song 2017 (75)	Female/49	CT Excisional biopsy Immunohistochemistry	Surgical resection	Clinically stable during follow-up
Jiyong Wu 2019 (74)	Male/67	CT Excisional biopsy Immunohistochemistry	Not reported	Not reported
Jikai Zhao 2019 (17)	Male/54	CT Excisional biopsy Immunohistochemistry	Surgical resection Chemotherapy (paclitaxel and carboplatin)	Clinically stable during follow-up

CT, computed tomography; PET-CT, positron emission tomography-CT.

PEC tumors can manifest in the bladder, uterus, breast, ovary, vulva, vagina, kidney, prostate, pancreas, jejunum and soft tissues, often with benign behavior (2, 14). PEC tumors originating in soft tissues and gynecological have some findings suggestive of malignancy (8), being indicative in the presence of 2 or more criteria: > 5 cm, infiltration, high-grade atypia, necrosis, vascular invasion, mitoses > 1/50 high power fields. Some authors (80) suggest greater specificity for PEC tumors of gynecological origin with at least 4 or more of these characteristics. But WHO followed the guidance of at least 3 criteria for malignancy (81).

There are currently no well-defined diagnostic methods for malignant CCTL. Zhao et al. proposed major and minor criteria for differentiating between benign and malignant manifestations (17). Imaging characteristics are nonspecific, and the tumor can appear in any lobe (although more commonly in the bilateral lower lobes) with a well-demarcated nodule without cavitation or calcification (24). The presence of a single and intraparenchymal pulmonary nodule is common to several diseases in addition to CCTL, such as bronchogenic carcinoma and oat cell carcinoma, others tumors such as angiomyolipoma, and with metastases from clear cell carcinoma of the kidney, breast, liver, ovary, uterus and cervix, adrenal and melanoma (28, 82). Due to the nonspecificity of the image, histopathological and immunohistochemical is highly recommended for the CCTL characterization (19)

At optical microscopy the CCTL cells are round or oval, have clear cytoplasm strongly stained by PAS due to the presence of glycogen, mild nuclear pleomorphism and a characteristic plexus of prominent sinusoidal vessels, but mitotic activity and necrosis are absent (19, 24). This characteristic pattern helps to differentiate CCTL from other conditions, such as angiomyolipoma and adrenal carcinoma (28, 82).

Tumor cells show reactivity for HMB-45 in about 90% of cases, similar to most mesenchymal tumors, and do not express cytokeratins (AE1/AE3, CK7, CK8 and CK20) and epithelial membrane antigen (EMA) in 90% of cases, a fact that facilitates differentiation from other types of tumors (18, 19, 28), as observed in our case. The immunoreactivity of renal cell carcinoma to EMA, cytokeratins, CD10 and RCC are useful for the differentiation of CCTL, despite having vimentin as a positive marker in common. However, in the case of atypical melanomas, positivity to HMB-45 and S-100 present in both, does not allow elucidation of the diagnosis (28). Cathepsin K, the main marker for differentiating PEC tumors from other neoplasms, was diffusely positive in 5 cases of CCTL described in the literature (82), demonstrating the superiority of this marker over HMB-45 in differentiating CCTL from other lung tumors. Thus, the co-expression of HMB-45 and cathepsin K markers ensures a more accurate diagnosis.

Furthermore, other markers such as S-100, vimentin, CD34, Melan-A, microphthalmia factor, neuron-specific enolase, cathepsin B, and CD1a can be expressed (18, 20, 28). CD10 may be expressed in other types of non-pulmonary PEComas (83, 84). Our patient tested positive for HMB-45, Melan-A, smooth muscle actin, and CD10, and tested negative for cytokeratins.

Complete surgical resection is probably the most effective therapeutic method at present (85). Radiotherapy may be considered for patients with surgical contraindications (20). Chemotherapy should be administered for malignant manifestations, as in the reported case. CCTLs upregulate the mammalian target of rapamycin (mTOR) signaling pathway; therefore, mTOR inhibitors can be used in such cases (86, 87).

Despite being described in the literature, mitotic activity and some biomarkers may not differ between malignant and benign CCTLs. Except for lesion size, clinical characteristics and complementary examinations cannot predict a worse development. Close follow-up can identify when CCTL cases may exhibit unexpected and malignant behaviors.

## Conclusion

Herein, we report the clinical evolution of a patient with malignant CCTL. Although this form of presentation is extremely rare in the literature, it is not yet possible to establish clinical, immunohistochemical, or histopathological criteria to predict the prognosis and possible malignant evolution. Although described in some studies, mitotic activity does not differentiate between benign and malignant tumors. The size of the initial lesion, presence of multiple lesions, and patient’s clinical evolution seem to be the only comparative parameters.

## Data availability statement

The original contributions presented in the study are included in the article/supplementary material. Further inquiries can be directed to the corresponding author.

## Ethics statement

Written informed consent was obtained from the proxy for the patient described in the manuscript.

## Author contributions

MC: Conceptualization, Methodology, Writing – review & editing. LV: Data curation, Formal Analysis, Investigation, Writing – original draft. RM: Data curation, Formal Analysis, Investigation, Writing – original draft. ÉM: Conceptualization, Methodology, Writing – original draft. AF: Conceptualization, Methodology, Writing – original draft. SS: Conceptualization, Methodology, Writing – original draft. GS: Supervision, Visualization, Writing – review & editing. MM: Data curation, Visualization, Writing – review & editing. MZ: Data curation, Investigation, Visualization, Writing – review & editing. DQ: Conceptualization, Investigation, Supervision, Writing – review & editing.
